# Synthesis of Chlorinated Tetracyclic Compounds and Testing for Their Potential Antidepressant Effect in Mice

**DOI:** 10.3390/molecules21010061

**Published:** 2016-01-05

**Authors:** Usama Karama, Mujeeb A. Sultan, Abdulrahman I. Almansour, Kamal Eldin El-Taher

**Affiliations:** 1 Chemistry Department, College of Science, King Saud University, P. O. Box 2455 Riyadh 11451, Saudi Arabia; Alhosami1983@yahoo.co.uk (M.A.S.); almansor@ksu.edu.sa (A.I.A.); 2 Department of Pharmacology, College of Pharmacy, King Saud University, P. O. Box 2457, Riyadh 11451, Saudi Arabia; eeltahir@ksu.edu.sa

**Keywords:** chlorinated tetracyclic, antidepressants, synthesis, forced swimming test

## Abstract

The synthesis of the tetracyclic compounds 1-(4,5-dichloro-9,10-dihydro-9,10-ethanoanthracen-11-yl)-*N*-methylmethanamine (**5**) and 1-(1,8-dichloro-9,10-dihydro-9,10-ethanoanthracen-11-yl)-*N*-methylmethanamine (**6**) as a homologue of the anxiolytic and antidepressant drugs benzoctamine and maprotiline were described. The key intermediate aldehydes (**3**) and (**4**) were successfully synthesized via a [4 + 2] cycloaddition between acrolein and 1,8-dichloroanthracene. The synthesized compounds were investigated for antidepressant activity using the forced swimming test. Compounds (**5**), (**6**) and (**3**) showed significant reduction in the mice immobility indicating significant antidepressant effects. These compounds significantly reduced the immobility times at a dose 80 mg/kg by 84.0%, 86.7% and 71.1% respectively.

## 1. Introduction

Depression is the most common psychiatric disorder and is among the 10 leading causes of morbidity and mortality worldwide [1,2]. Recent studies have indicated that depression occurs in all ethnic, racial, and socioeconomic groups and can occur at any age, from childhood to old age. These studies concluded that depression is a common psychiatric disorder in the general population. On an international scale, prevalence estimates of depression are consistently high; varying with population studied and study design. Depression affects more than 340 million people globally [3].

Many of the most common drugs and drug candidates contain halogens. Halogen atoms are often introduced in order to increase lipophilicity, binding affinity and membrane permeability, to fill hydrophobic cavities in the protein binding site, to facilitate the blood-brain barrier crossing and to prolong the lifetime of the drug thereby improves bioavailability [4,5,6,7]. All the selective serotonin reuptake inhibitor (SSRI) antidepressants such as citalopram, escitalopram, fluoxetine, fluvoxamine, paroxetine, and the very effective top-selling antidepressant drug sertraline possess halogen atoms at specific positions , which are key determinants for the drugs specifity for serotonin transporter (SERT) [7].

The tetracyclic drugs benzoctamine 1-(9,10-dihydro-9,10-ethanoanthracen-9-yl)-*N*-methylmethanamine and maprotiline 3-(9,10-dihydro-9,10-ethanoanthracen-9-yl)-*N*-methylpropanamine are bridged anthracene compounds that do not possess halogen atoms [8]. Despite their structural similarity, benzoctamine is primarily an anxiolytic drug that exhibits antagonistic effects on norepinephrine, while maprotiline is an antidepressant and anxiolytic drug that is useful as a norepinephrine uptake inhibitor. Seizures, leukopenia and skin reactions are common side effects of maprotiline. Thus, antidepressants compounds solving the aforementioned problems are desired. Based on the the above evidence, it was decided to synthesis the novel chlorinated maprotiline analogues (**5**) and (**6**), since the insertion of a chlorine atoms in the maprotiline related compounds could be a small change for a big improvement aiding in the development of more potent analogues.

## 2. Results and Discussion

### 2.1. Chemistry

**Scheme 1:**
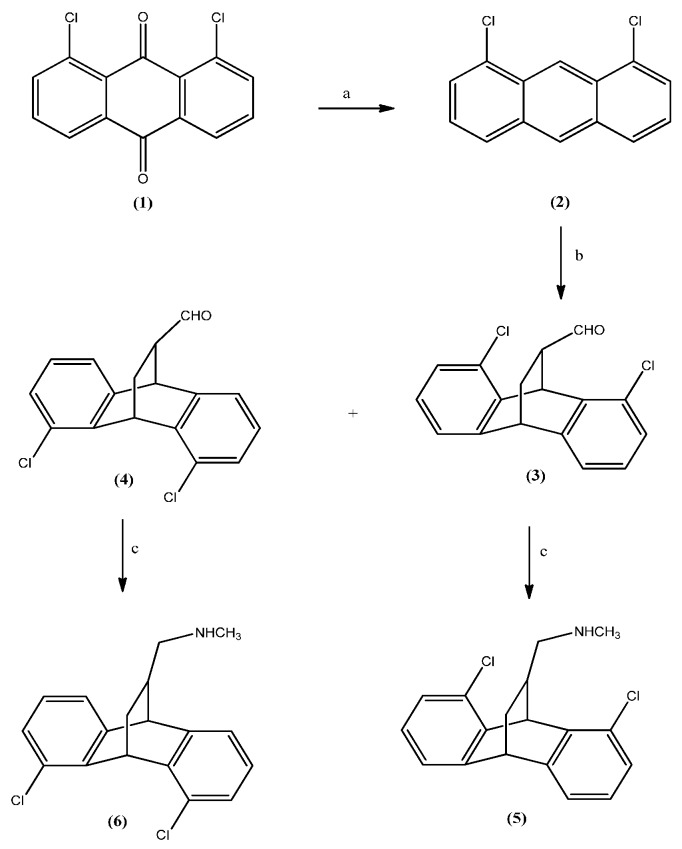
Synthesis of compounds (**5**) and (**6**). *Reagents and conditions*. (a) Zn, NH_3_/H_2_O, 100 °C, 3 h; HCl, isopropanol, 100 °C , 3 h, 62%; (b) acrolein, CH_2_Cl_2_, BF_3_·OEt_2_, r. t., 3 h, 76%; (c) H_2_, Pd/C, NH_2_CH_3_, CH_3_OH, r. t., 4 h, 85%.

**Figure 1:**
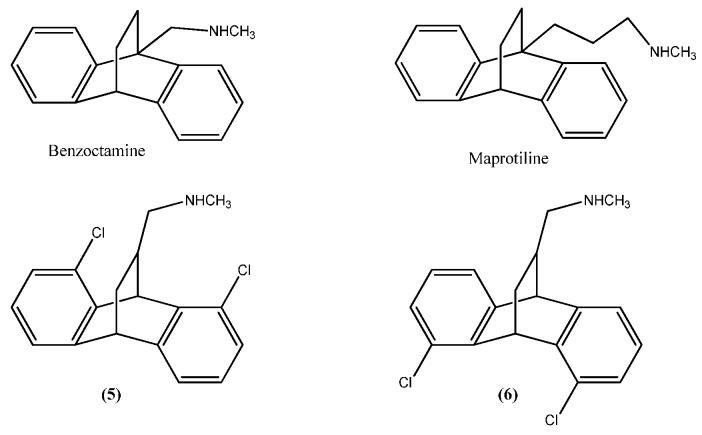
Benzoctamine, maprotiline and targeted analogues.

We outline a simple, flexible and economical synthetic route (Scheme 1) to the corresponding 1-(4,5-dichloro-9,10-dihydro-9,10-ethanoanthracen-11-yl)-*N*-methylmethanamine (**5**) and 1-(1,8-dichloro-9,10-dihydro-9,10-ethanoanthracen-11-yl)-*N*-methylmethanamine (**6**) (Figure 1) [9]. The key step was Diels-Alder [4 + 2] cycloaddition reaction at room temperature in the presence of a catalytic amount of boron trifluoride etherate between acrolein and 1,8-dichloroanthracene (**2**), which was obtained according to the literature procedure by the reduction of the commercial available 1,8-dichloroanthraquinone (**1**) with zinc powder in aqueous ammonia followed by an acidic treatment [10]. The two isomeric cycloadducts (**3**) and (**4**) were separated by column chromatography and were converted to the desired amines (**5**) and (**6**) by direct reductive amination of the corresponding aldehyde.

### 2.2. Assessment of Antidepressant Activity Using Forced Swimming Test

Table 1 shows the Effect of compounds (**3**), (**4**), (**5**) and (**6**) on forced swimming test in mice—a model for testing antidepressant activity. As shown in Table 1; compounds (**3**), (**5**) and (**6**) all at doses of 80 mg/kg produced significant decrease in immobility time (*p* < 0.05, *n* = 4) compared with control vehicle and maprotiline. Compound **5** and **6** at the same dose were significantly more active than compound (**3**) (*p* < 0.05, *n* = 4). Only compounds (**3**), and (**5**) were significantly active in reducing immobility time at doses of 40 mg/kg compared with control vehicle (*p* < 0.05, *n* = 4). Compound (**4**) was completely inactive in this test at all doses tested. Table 2 shows the percentage decrease in immobility times induced by each dose compared with control vehicle.

**Table 1:** Effects on Immobility Duration.

Animal Group	Duration of Immobility in Seconds during the Last Four Minutes of the Six Minute Period				
	0 mg/kg	10 mg/kg	20 mg/kg	40 mg/kg	80 mg/kg
Control vehicle	225 ± 7				
Maprotiline		157 ± 5	156 ± 4	144 ± 5	134 ± 5
Treated with (**3**)		240	240	160±11 ^b^	65 ± 4 ^a,b^
Treated with (**4**)		240	240	240	240
Treated with (**5**)		240	200 ± 12.3	140 ± 7.9 ^b^	36 ± 4.9 ^a,b^
Treated with (**6**)		240	240	240	30 ± 5 ^a,b^

All values represent mean ± SEM. ^a^ *p* < 0.05, *n* = 4 compared with maprotiline, ^b^ *p* < 0.05, *n* = 4 compared with control.

**Table 2:** Percentage decrease in Immobility Times.

Compound	Percentage Decrease for Dosage of 40 mg/kg	Percentage Decrease for Dosage of 80 mg/kg
**(3)**	28.9	71.1
**(5)**	37.8	84.0
**(6)**	-	86.7

## 3. Experimental

### 3.1. General Protocol

IR spectra were recorded on a Perkin-Elmer 883 spectrophotometer and peaks are expressed as ν (cm^−1^). NMR spectra were recorded on a JEOL ECP 400 (400 MHz) instrument in CDCl_3_ and chemical shifts are expressed as δ ppm, and coupling constants (*J*) are given in Hertz. MS spectra and HRMS were performed at the Department of Organic Chemistry of the University of Hannover, Germany using EI at 70 eV.

### 3.2. Synthesis

*1,8-Dichloroanthracene* (**2**). A suspension of 1,8-dichloroanthraquinone (**1**) (10.0 g, 36.1 mmol) and zinc dust (50.0 g, 765 mmol) in (200 mL) of aqueous 28% NH_3_ was stirred for 3 h at 100 °C. After cooling to room temperature, the resulting solid was separated by suction filtration. A CH_2_Cl_2_ solution of the resulting crude solid was combined with the CH_2_Cl_2_ extract of the supernatant liquid and the mixture dried over MgSO_4_ and concentrated under vacuo. The residual crude solid was dissolved in a mixture of (500) mL of isopropanol and (50) mL of aqueous 12 M HCl and the resulting solution had been refluxed for 3 h, then concentrated and partitioned between CH_2_Cl_2_ and aqueous 5% NaHCO_3_. The organic layer was collected, dried over MgSO_4_. The solvent was evaporated and the crude solid product was recrystallized from a CH_2_Cl_2_–hexane mixture. The collected product was allowed to dry in the air for 24 h to provide (5.5 g, 62%) of 1,8-dichloroanthracene (**2**) as yellow needles: mp 153 °C; IR (KBr): ν = 1617, 1547, 1438, 1300, 1210, 953, 872, 775, 720, 678 cm^−1^; ^1^H-NMR (CDCl_3_, 400 MHz) δ = 7.25–7.88 (m, 6 H, ArH), 8.36(s, 1 H, ArH-10), 9.16 (s, 1 H, ArH-9); ^13^C-NMR (CDCl_3_, 100 MHz) δ = 120.9, 125.6, 126.0, 127.3, 127.6, 129.2, 132.4, 132.6; MS (EI) *m*/*z* (%) = 246 (100) [M]^+^, 248 (65); HRMS (EI) Calcd. For C_14_H_8_C_l2_ [M]^+^ 246.0003, Found 246.0001.

*4,5-Dichloro-9,10-dihydro-9,10-ethanoanthracene-11-carbaldehyde* (**3**)

*1,8-Dichloro-9,10-dihydro-9,10-ethanoanthracene-11-carbaldehyde* (**4**)

Acrolein (1.65 mL, 23.8 mmol) was added to a solution of 1,8-dichlorolanthracene (**2**) (1.23 g, 5 mmol) in (70 mL) CH_2_Cl_2_ followed by dropwise addition of BF_3_·OEt_2_ (0.63 mL, 5 mmol). The mixture was stirred at room temperature for 3 h. During this time the solution gradually turned brown, then the reaction was quenched with brine and extracted 3 times with CH_2_Cl_2_. The organic layer was collected and dried over Na_2_SO_4_ and the solvent was removed in vacuo. Column chromatography of the residue on silica gel (ethyl acetate/petroleum ether, 1:10) yields the aldehyde (**3**) (R_f_ = 0.41) as colorless oil (0.15 g, 10%) and the aldehyde (**4**) (R_f_ = 0.30) as colorless oil (1.0 g, 66%).

(**3**) IR (KBr): ν = 2924, 1725, 1576, 1435, 1260, 1167, 1046, 770, 704 cm^−1^; ^1^H-NMR (CDCl_3_, 400 MHz): δ = 2.01–2.04 (m, 1H, H-12), 2.10–2.12 (m, 1H, H-12), 2.81 (m, 1-H, H-11), 4.42 (t, *J* = 2.6, 1H, H-9), 5.71 (d, *J* = 2.5, 1H, H-10), 7.10–7.36 (m, 6 H, ArH), 9.43 (d, *J* = 1.7, 1 H, CHO); ^13^C-NMR (CDCl_3_, 100 MHz): δ = 27.8, 37.8, 44.3, 50.6, 122.2, 126.7, 127.6, 127.9,129.7, 136.2, 138.4, 145.7, 146.1, 200.7; MS (EI) *m*/*z* (%) = 302 (20) [M]^+^, 248 (85), 246 (100), 211 (5), 178 (4); HRMS (EI) Calcd. For C_17_H1_2_OCl_2_ [M]^+^ 302.0265, Found 302.0266.

(**4**) IR (KBr): ν = 2944, 1727, 1577, 1455, 1210, 1167, 785, 770 cm^−1^; ^1^H-NMR (CDCl_3_, 400 MHz): δ = 2.04–2.07 (m, 1H, H-12), 2.11–2.12 (m, 1-H, H-12), 2.79 (m, 1H, H-11), 4.75 (d, *J* = 2.5, 1H, H-10), 5.48 (t, *J* = 2.7, 1H, H-9), 7.04–7.28 (m, 6 H, ArH), 9.46 (d, *J* = 1.4, 1 H, CHO); ^13^C-NMR (CDCl_3_, 100 MHz): δ = 26.9, 36.6, 36.7, 45.5, 50.5, 122.1, 123.2, 127.1, 127.3, 129.7, 130.0, 141.5, 144.1, 201.7; MS (EI) *m*/*z* (%) = 302 (20) [M]^+^, 248 (75), 246 (100), 212 (8), 178(78); HRMS (EI) Calcd. For C_17_H_12_OCl_2_ [M]^+^ 302.0265, Found 302.0265.

*1-(4,5-Dichloro-9,10-dihydro-9,10-ethanoanthracen-11-yl)-N-methylmethanamine* (**5**). In two-necked round-bottomed flask (40 mg 10% Pd/C) was wetted with methanol and the flask was evacuated, then flushed with hydrogen two times, then a solution of (100 mg, 0.33 mmol) aldehyde (**3**) in (5 mL) methanol was added to the reaction mixture followed by the addition of (0.5 mL, 2 M) solution of methylamine in methanol. The mixture was stirred for 4 hours at room temperature under H_2_ atmosphere (balloon). The reaction mixture was filtered through a pad of celite and the solvent was removed in vacuo to yield (90 mg, 85%) of the corresponding amine **5** as white powder. mp 290 °C; IR (KBr): ν = 3440, 2942,2864, 2775, 1592, 1457, 1410, 1026, 936, 755, 742, 555 cm^−1^; ^1^H-NMR (CDCl**_3_**, 400 MHz) δ = 1.27–1.37( m, 1H, H-12 ), 2.13–2.19 (m, 1H, H-12), 2.47–2.50 (m, 1H, H-11), 2.55–2.66 (m, 5H, CH_2_-N-CH_3_), 4.31 (t, *J* = 2.5, 1H, H-9), 4.63 (d, *J* = 2.0, 1H, H-10), 6.98–7.05 (m, 3 H, ArH), 7.17–7.19 (m; 2 H, ArH), 7.45–7.48 (m; 1 H, ArH); ^13^C-NMR (CDCl**_3_**, 125 MHz) δ = 33.2, 33.9, 35.9, 43.7, 46.0, 54.7, 123.3, 123.5, 123.8, 125.6, 125.9, 126.0, 126.4, 139.3, 142.7, 142.9, 143.5. MS (EI): *m*/*z* (%) = 317 [M]^+^ (not recorded), 306 (7), 305 (22), 251 (75), 250 (100), 220 (7), 219 (22), 179 (5), 179 (4), 178 (13), 111 (8).

*1-(1,8-Dichloro-9,10-dihydro-9,10-ethanoanthracen-11-yl)-N-methylmethanamine* (**6**). In two-necked round-bottomed flask (40 mg 10% Pd/C) was wetted with methanol and the flask was evacuated, then flushed with hydrogen two times, then a solution of (100 mg, 0.33 mmol) aldehyde (**4**) in (5 mL) methanol was added to the reaction mixture followed by the addition of (0.5 mL, 2 M) solution of methylamine in methanol. The mixture was stirred for 4 h at room temperature under H_2_ atmosphere (balloon). The reaction mixture was filtered through a pad of celite and the solvent was removed in vacuo to yield (90 mg, 85%) of the corresponding amine (**6**) as white powder. mp 310 °C; IR (KBr): ν = 3435, 2943, 2774, 1626, 1593, 1457, 1411, 1027, 937, 758, 742, 555 cm^−1^; ^1^H-NMR (CDCl_3_, 500 MHz) δ = 1.24–1.26 (m, 1H, H-12), 2.04–2.08 (m, 1H, H-12), 2.38–2.40 (m, 1H, H-11), 2.45–2.54 (m, 5H-CH_2_-N-CH_3_), 4.21 (broad s, 1H, H-10), 4.52 (broad s, 1H, H-9), 6.98–7.05 (m, 3H, ArH), 7.17–7.19 (m, 2H, ArH), 7.42–7.43 (m, 1H, ArH); ^13^C-NMR (CDCl_3_, 125 MHz) δ = 33.1, 33.6, 35.5, 43.5, 45.8, 54.3, 123.4, 123.5, 123.8, 125.6, 126.0, 126.1, 126.5, 139.1, 142.4, 142.8, 143.4; MS (EI) *m/z* (%) = 318 (100) [M + H]^+^, 284 (22), 186(15), 117 (53); HRMS (EI) Calcd. for C_18_H_18_NCl_2_ [M]^+^ 318.0816, Found 318.0809.

### 3.3. Assessment of Antidepressant Activity Using Forced Swimming Test

To test for the presence of anti-depressant activity of the test compounds (**3**), (**4**), (**5**) and (**6**). The forced swimming test described previously by David *et al.* [11] was used with very slightly modification regarding the dimension of the vessel used as a swimming pool. This vessel consisted of a glass cylinder having an internal diameter of 25 cm and water depth of 15 cm and maintained at 22 ± 2 °C. Swiss white mice weighing 25 g each were divided into 21 groups (*n* = 4). One group served as control vehicle and the others divided for each test compound and maprotiline.

The test compounds and maprotiline were dissolved in DMSO and examined at 4 dose levels 10, 20, 40, 80 mg/kg (i.p.). The compounds and maprotiline were administered 20 min before starting the swimming test. The control group was administered the vehicle only in the same volume. Each mouse was allowed to stay in the swimming pool for 6 min. Mice normally swim for 1–2 min and then turn helpless and become immobile. An immobile mouse is considered the one that does not show any swimming attempt but shows only movements that allows it to keep its head above the surface of the water. Counting of the time of immobility started at the beginning of the third minutes of placing the mouse into the swimming pool and ended by the end of the sixth minute. In this test antidepressant drugs decrease time of immobility. Thus, for each test compound dose, the time of immobility was calculated. The means ± SEM values were calculated for each group. The data were analyzed using one-way ANOVA followed by Dunnet’s multiple comparison test. *p* < 0.05 was considered to be statistically significant.The percentage decreases in immobility times induced by each doses were calculated.

## 4. Conclusions

In conclusion, novel chlorinated tetracyclic compounds were successfully synthesized as analogues of benzoctamine and maprotiline by a simple, economical and flexible three-step formal synthesis from simple and cheap starting materials. The key cyclisation step was accomplished through the Diels-Alder reaction at room temperature. The two target compounds proved to possess potential antidepressant activity as tested by the forced swimming test in mice.

## References

[1] González H.M., Tarraf W., Whitfield K.E., Vega W.A. (2010). The epidemiology of major depression and ethnicity in the united states. J. Psychiatr. Res..

[2] McKenna M.T., Michaud C.M., Murray C.J., Marks J.S. (2005). Assessing the burden of disease in the united states using disability-adjusted life years. Am. J. Prev. Med..

[3] Collins K.A., Fitterling H.L. (2009). Physical exercise and depression. Mt. Sinai J. Med..

[4] Parisini E., Metrangolo P., Pilati T., Resnati G., Terraneo G. (2011). Halogen bonding in halocarbon–protein complexes: A structural survey. Chem. Soc. Rev..

[5] Erdelyi M. (2012). Halogen bonding in solution. Chem. Soc. Rev..

[6] Kolář M., Hobza P., Bronowska A.K. (2013). Plugging the explicit σ-holes in molecular docking. Chem. Commun..

[7] Liu Y., Xu Z., Yang Z., Chen K., Zhu W. (2013). A knowledge-based halogen bonding scoring function for predicting protein-ligand interactions. J. Mol. Model..

[8] Wilhelm M., Schmidt P. (1969). Synthese und eigenschaften von 1-aminoalkyl-dibenzo[*b*,*e*]bicyclo[2.2.2]octadienen. Helv. Chim. Acta.

[9] Karama U.S., Sultan M.A.S., EL-Tahir K.E.H., Almansour A.I. (2015). Antidepressant Compounds. U.S. Patent.

[10] House H.O., Ghali N.I., Haack J.L., van Derveer D. (1980). Reactions of the 1,8-diphenylanthracene system. J. Org. Chem..

[11] David D.J.P., Renard C.E., Jolliet P., Hascoët M., Bourin M. (2003). Antidepressant-like effects in various mice strains in the forced swimming test. Psychopharmacology.

